# Jejunal Mesenteric Well-Differentiated Liposarcoma With Isolated Omental Metastases: Evidence of Focal Dedifferentiation

**DOI:** 10.7759/cureus.107186

**Published:** 2026-04-16

**Authors:** Shrikant Yadav, Vipul K Srivastava, Harsh V Baranwal, Anup K Pandit, Mantu Yadav, Sangeeta Kumari, Sanjay K Saroj, Satyanam K Bhartiya

**Affiliations:** 1 General Surgery, Institute of Medical Sciences, Banaras Hindu University (BHU), Varanasi, IND

**Keywords:** dedifferentiated liposarcoma, jejunal mass, liposarcoma, mesenteric liposarcoma, mesenteric tumour, omental metastasis, soft tissue sarcoma

## Abstract

A malignant tumor of adipocytic origin, liposarcoma typically affects the deep soft tissues and retroperitoneum. Although primary mesenteric liposarcoma is uncommon, its association with isolated omental metastasis is a rare entity. A 65-year-old male presented with a painless, progressively enlarging abdominal mass. Imaging revealed multiple fat-attenuated intra-abdominal lesions. Surgical exploration confirmed a large lobulated mesenteric mass and an omental mass. Wide local excision with omentectomy and jejuno-jejunal anastomosis was performed. Histopathology confirmed well-differentiated liposarcoma with focal areas of dedifferentiation with omental metastasis. The patient had an uneventful postoperative recovery and was referred for adjuvant therapy. Jejunal mesenteric liposarcoma with omental metastasis is a rare entity. Prompt diagnosis and complete surgical resection with negative margins are the mainstay of management. Long-term surveillance is required due to recurrence risk.

## Introduction

Liposarcomas are rare malignant tumors of mesenchymal origin, most commonly found in the retroperitoneum. They account for approximately 20% of all adult soft tissue sarcomas and may remain asymptomatic until they reach a significant size due to the expansile capacity of the abdominal cavity [1]. Primary mesenteric liposarcoma is exceedingly rare, representing less than 1% of all intra-abdominal tumors. Among the histological subtypes, well-differentiated liposarcoma (WDL) is relatively indolent but carries a risk of recurrence and metastasis [2]. Metastatic spread from a predominantly well-differentiated mesenteric liposarcoma is exceptionally uncommon and should prompt consideration of dedifferentiated transformation. We present a case of a large mesenteric liposarcoma with isolated omental metastasis presenting as abdominal swelling, managed surgically.

## Case presentation

A 65-year-old male presented with a chief complaint of progressive abdominal swelling for three months. There was no history of pain, fever, jaundice, vomiting, altered bowel habits, melena, or urinary complaints. His past history was unremarkable with no comorbidities or prior surgeries.

Clinical examination revealed a large intra-abdominal, non-tender, firm, ill-defined lump (15 × 20 cm) with side-to-side mobility, covering almost the whole of the abdomen. A second lump (5 × 4 cm) was noted in the right iliac fossa. There was no ascites or free fluid. Bowel sounds were heard normally. The biochemical parameter was within normal limits.

**Figure 1:**
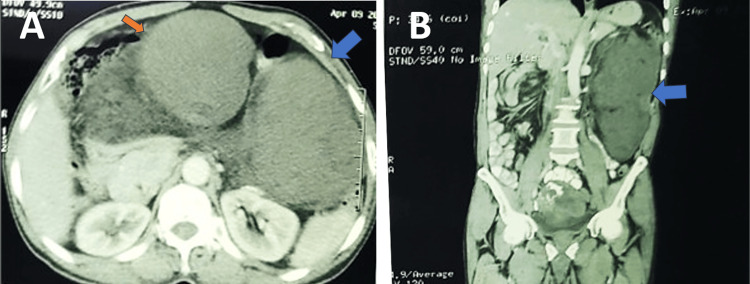
(A) Axial section of CT abdomen showing the tumor with the surrounding structures. Orange and blue arrows indicate lobules of the lesion. (B) Coronal section of CT abdomen showing the tumor (blue arrow) and its relation with the surrounding structures.

Contrast-enhanced computed tomography (CECT) of the abdomen revealed a large, multi-lobulated, 60 to 160 mm well-defined mass lesion in the peritoneal cavity at the abdominopelvic region, with a few of them showing areas of fat attenuation with calcifications, likely sarcoma or liposarcoma (Figure 1). Another lesion of size 5 × 5 cm was noted in the right iliac fossa, closely abutting the common iliac vessels.

**Figure 2:**
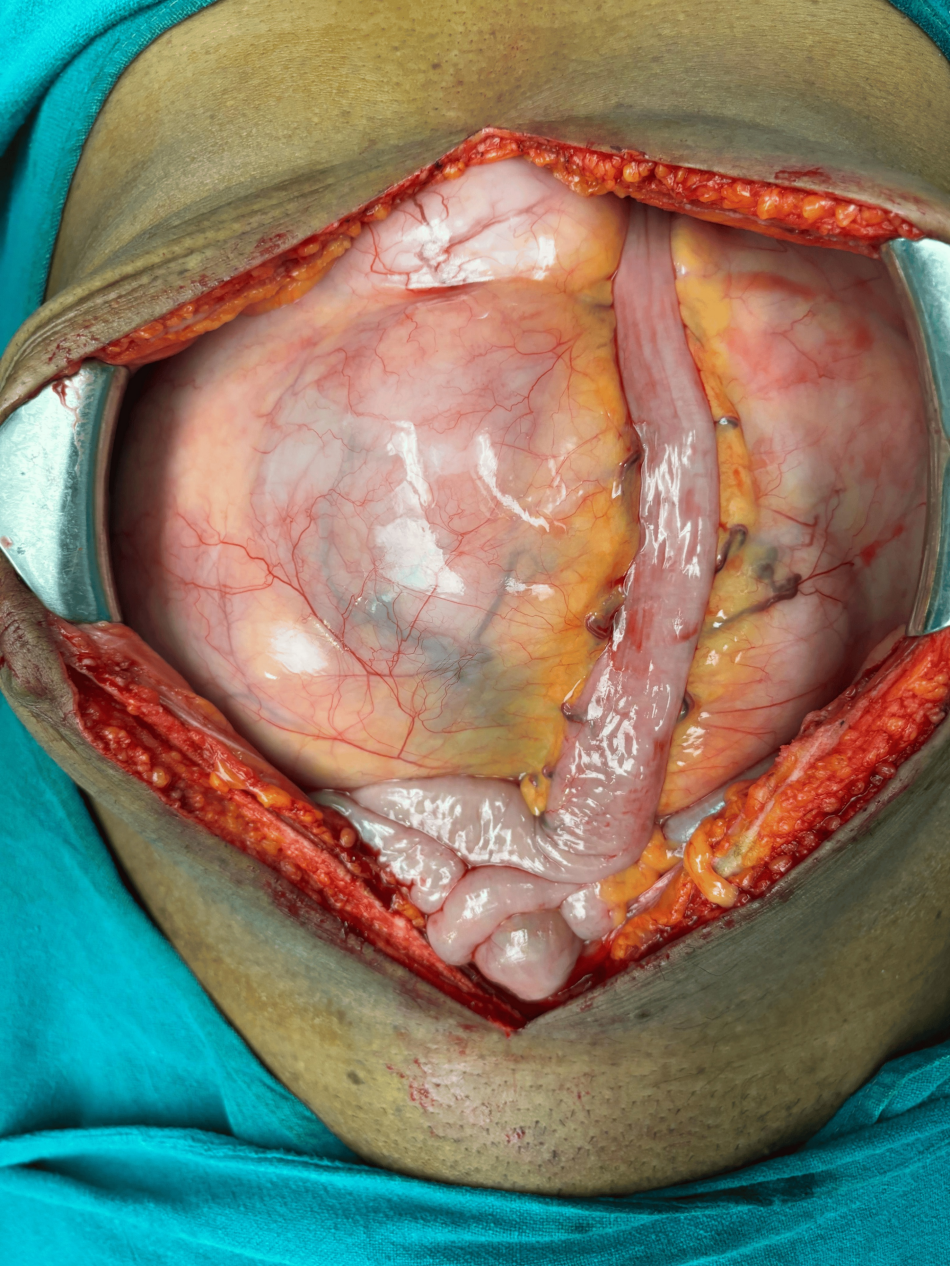
Intra-operative image of the tumor.

**Figure 3:**
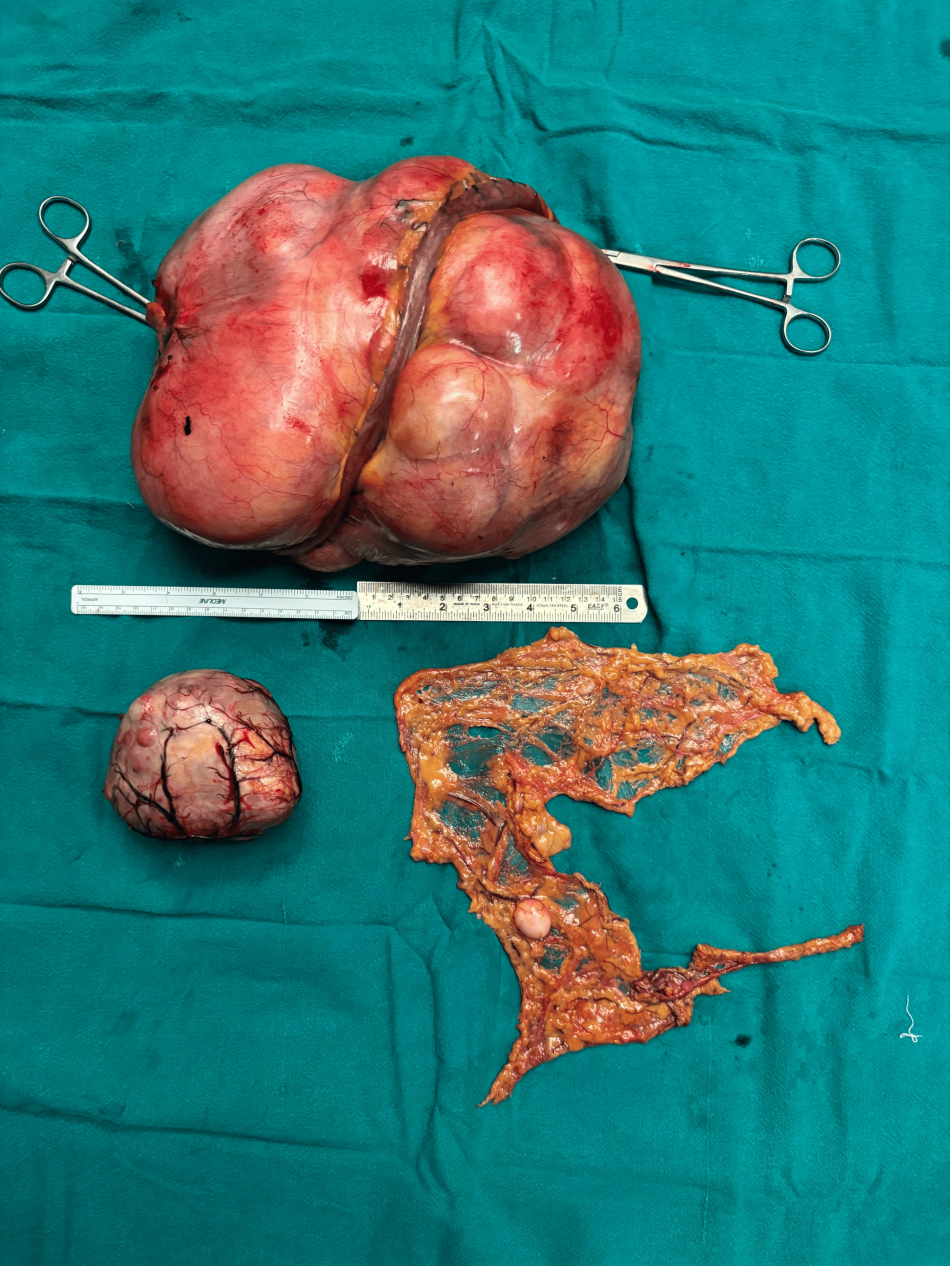
Resected jejunal mesenteric mass with small bowel, resected omental deposits, and omentum.

The patient underwent an exploratory laparotomy, which revealed a 25 × 22 × 14 cm jejunal mesenteric mass and a 10 × 8 cm omental mass. Two to three other similar but smaller lesions were seen in the omentum without any evidence of metastases into other organs or peritoneum. En bloc resection of the jejunal mesenteric mass along with omentectomy and end-to-end jejunojejunal anastomosis was performed (Figures 2-3). Postoperatively, the patient had an uneventful recovery. He passed flatus and stool by postoperative day 4, was started on oral intake on day 5, and was discharged in a stable condition.

**Figure 4:**
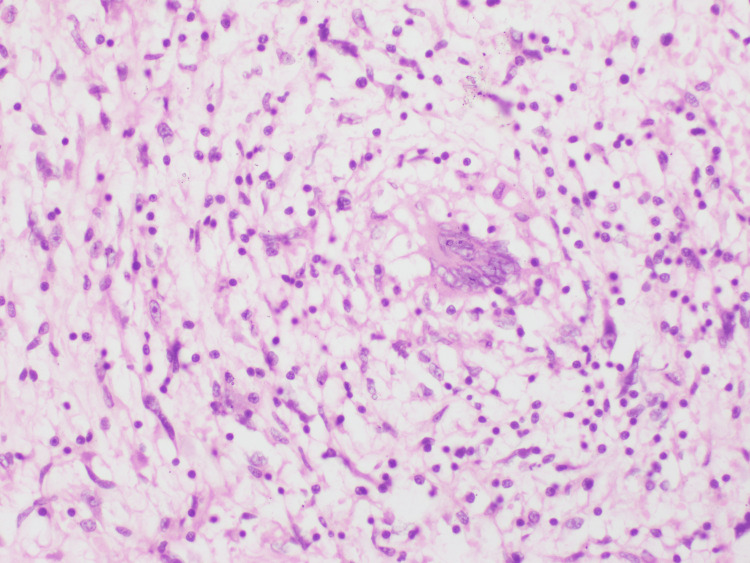
Histopathology(H&E, ×40) showing a tumor composed of large areas of adipocyte clusters with fibroblasts, lipoblasts, and proliferation of stromal cells. Cells are long, elongated, oval-shaped, and spindle in nature, with hyperchromatic pleomorphic nuclei.

Histopathological examination of the resected jejunal mesenteric mass with small bowel showed a tumor composed of large areas of adipocyte clusters with fibroblasts, lipoblasts, and proliferation of stromal cells, suggestive of well-differentiated soft tissue sarcoma (Figure 4). Areas of dedifferentiation identified comprising sheets and fascicles of spindle cells with markedly pleomorphic nuclei, and immunohistochemistry demonstrated CDK4 amplification with non-contributory Murine double minute 2 (MDM2) (Table 1). Stroma shows myxoid changes. Both margins of resection were free of tumor. The tumor reaches up to the serosa of the bowel wall. Omentum shows metastatic tumor depositions.

**Table 1:** Immunohistochemistry (IHC) of the postoperative specimen. CD117 - cluster of differentiation; CDK4 - cyclin-dependent kinase 4; MDM2 - murine double minute 2; SMA - smooth muscle actin

IHC Marker	Result
CDK4	Amplified
MDM2	Non-contributory
SMA	Negative
Desmin	Negative
S-100	Negative
CD-117	Negative

After multidisciplinary tumor board review, the patient was referred for adjuvant systemic chemotherapy and was treated with five cycles of epirubicin- and ifosfamide-based chemotherapy. He was subsequently followed under routine sarcoma surveillance protocol with periodic clinical assessment and serial CECT imaging. At one-year follow-up, the patient remained asymptomatic with no radiological evidence of disease recurrence or metastatic progression.

## Discussion

Liposarcomas are malignant tumors of adipocytic origin and represent approximately 20% of all adult soft tissue sarcomas [1]. They are most frequently found in the retroperitoneum and deep soft tissues of the extremities, but are extremely rare in the mesentery. Mesenteric liposarcomas account for less than 1% of intra-abdominal tumors, with even fewer cases involving omental metastasis [1-3].

The clinical presentation of mesenteric liposarcoma is typically non-specific and dominated by symptoms related to mass effect, such as abdominal distension or a palpable lump, rather than systemic signs. In our case, the patient presented with progressive, painless abdominal swelling over three months, similar to other reported cases [2,4].

CECT plays a vital role in diagnosis, with liposarcomas appearing as large masses that may exhibit internal septations or calcifications. Imaging alone, however, cannot reliably distinguish between benign lipomas and WDLs, necessitating histopathological evaluation [1,5]. Our case had typical imaging features consistent with sarcomatous transformation.

Histologically, WDLs are characterized by mature adipocytes with nuclear atypia and scattered lipoblasts, and they often express MDM2 amplification, a feature useful for diagnosis, particularly in differentiating WDL from benign lipomas [6]. In our case, the tumor showed classic features of WDL, with omental involvement - a rare event that is generally associated with dedifferentiated or high-grade variants [3,7]. The presence of focal dedifferentiated areas along with CDK4 amplification in our case provides a plausible biological explanation for the metastatic behavior, which would otherwise be atypical for a purely WDL. 

Ahire et al. and Yuri et al. describe similar cases of mesenteric liposarcoma but without metastatic spread [1,2]. However, our case exhibited omental deposits, raising the concern for an unusually aggressive clinical behavior despite well-differentiated histology. Similar metastatic patterns were reported by others, who documented cases of mesenteric dedifferentiated liposarcoma with peritoneal seeding, underscoring the potential for even WDL to present atypically [7,8].

The mainstay of treatment for liposarcoma remains wide surgical excision with negative margins [9]. In our case, we could have gone for palliative resection to extirpate the mass effect caused, but upon exploration, we did not find any other metastatic deposits except for the omentum. Thus, we decided to go with excision of the omental deposit along with omentectomy to reduce tumor burden. Adjuvant therapy is controversial in WDL, but may be considered in cases with dedifferentiation, positive margins, or metastasis [10]. Given metastatic disease and dedifferentiated features, adjuvant chemotherapy was administered in our case.

Long-term surveillance is critical due to the potential for local recurrence and late metastasis. In a retrospective series by Dalal et al., local recurrence of WDL occurred in up to 40% of patients, often many years after initial treatment [11].

## Conclusions

Mesenteric liposarcoma with isolated omental metastasis is an exceptionally rare clinical entity. This case highlights that tumors with predominantly well-differentiated morphology may harbor focal dedifferentiated components, which can account for atypical metastatic behavior. Complete surgical excision with negative margins remains the cornerstone of management, while selected high-risk cases may benefit from adjuvant systemic therapy. Given the risk of recurrence and aggressive biological potential, multidisciplinary management and long-term surveillance are essential for optimal outcomes.
